# Patient Satisfaction With Medical and Social Concerns Addressed During Telemedicine Visits

**DOI:** 10.7759/cureus.32529

**Published:** 2022-12-14

**Authors:** Heather N Abraham, Candace Acuff, Brittany Brauer, Renieh Nabaty, Ijeoma N Opara, Diane L Levine

**Affiliations:** 1 Internal Medicine-Pediatrics, Wayne State University School of Medicine, Detroit, USA; 2 Medicine, Wayne State University School of Medicine, Detroit, USA; 3 Internal Medicine, Wayne State University School of Medicine, Detroit, USA

**Keywords:** telemedicine patient satisfaction, social determinants of health (sdoh), virtual care delivery, covid-19, primary care, telemedicine, patient satisfaction with telehealth

## Abstract

Purpose

The use of telemedicine dramatically increased during the COVID-19 pandemic. Assessing patient satisfaction with this mode of healthcare delivery is an important metric of success as it is broadly implemented across various settings. Of additional importance are the ways social determinants of health impact health outcomes, with the first step in determining the scale of this impact being the identification of contributing factors. This study assesses patient satisfaction with the medical and social aspects of the care they receive via telemedicine at a university-affiliated primary care training clinic in Detroit, Michigan.

Methods

A survey was designed to assess patient satisfaction with the technical aspects of the visit, the visit itself, and with the social determinants screening tool used. During July 2020, 167 patients who had at least one telemedicine visit with a primary care physician from the clinic in the preceding months were contacted to assess their impression of the service provided. The responses were used to evaluate patient satisfaction with the comprehensive care provided via the telemedicine visit.

Results

Of the 167 patients contacted, 79 (47%) completed the survey. Respondents' age ranged from 18-74 years, with 66% identifying as female and 34% as male. For many, this was their first experience with telemedicine. The vast majority expressed comfort in sharing details about their health concerns via telemedicine, with only 3% reporting they were “uncomfortable.” More than half of the patients (60%) felt some level of comfort with telemedicine after their first encounter; 14% stated that they were still uncomfortable, and 26% were neutral. Most of the patients (88%) asserted their willingness to participate in future telemedicine visits. Just under two-thirds (63%) of participants “strongly agreed” that concerns related to their social determinants of health were addressed, and 59% “strongly agreed” that the resources provided by their physician were helpful.

Conclusion

This survey evaluates multiple dimensions of patient satisfaction with their physician using technology to deliver a telemedicine visit instead of an in-office visit. Telemedicine was well received, with high satisfaction for addressing medical and social concerns. The results of this study support the use of telemedicine to assess social determinants of health in an underserved minoritized patient population and will help physicians optimize future interactions with patients through telemedicine.

## Introduction

At the time of our study, the City of Detroit had many confirmed cases of the novel coronavirus, known as COVID-19, the most of any city in the State of Michigan, with nearly 10% of those resulting in death [1]. To maintain care while protecting patients and healthcare workers, a swift pivot to service delivery via telehealth was undertaken [2]. With national support from the American Medical Association (AMA) and other organizations, nearly all states relaxed their licensing rules allowing private insurance coverage of telemedicine. This included allowing coverage of audio-only services, waiving co-pays and co-cost sharing or requiring cost sharing no higher than in-person services, and requiring reimbursement parity between telemedicine and in-person services. All these factors contributed to the rise of the use of telemedicine starting in the early days of the pandemic [3]. 

Telemedicine facilitated the continuity of care for patients who would have been otherwise limited in their ability to access a physician due to the coronavirus. The advantages of telemedicine do not entirely supplant the value of face-to-face visits; however, patients and physicians acknowledge that these visits are not appropriate for all health-related topics [4]. Commonly cited patient concerns with telemedicine visits are the lack of in-person interaction and the resultant possibility for stilted rapport, with some worrying that the lack of in-person care may result in patient discomfort [5]. Difficulties with telemedicine historically cited by physicians and patients alike include limitations in physical exams, difficulty ordering appropriate testing, and mental health assessments. Privacy concerns related to information shared through online visits have also been raised [4]. 

Optimizing patient satisfaction is important to medical practitioners and healthcare centers. In previous studies, convenience, ease-of-use, decreased wait times, and reduced transportation costs had been cited as benefits of telemedicine visits to patients [4,6]. Advantages for patients specific to the pandemic included convenient access to physicians regardless of patient/physician location, resulting in expanded access to care, shorter wait times, and symptom screening to decrease transmission of COVID-19 [7,8]. Not only must telemedicine be of value to patients, but to be truly successful, healthcare providers must appreciate the use of technology for the delivery of their services. A study by Glaser et al. showed that 87% of clinicians who completed a telemedicine visit were satisfied with the visit, and 83% believed that the patient was also satisfied with the care they received, though the study did not specifically assess patient satisfaction [9]. With the increased utilization of telemedicine since the onset of the pandemic, multiple patient satisfaction studies have been reported. One systematic review analyzed 53 studies across 21 different specialties from January 2020 to August 2021. A common thread through most of the studies was the overall high patient satisfaction with telemedicine services [10]. There is, however, a literature deficit in addressing the social needs of patients via the virtual format. A study conducted by Abraham et al., which assessed third-year medical student satisfaction with their telehealth experiences during the COVID-19 pandemic, found that telemedicine training was an important addition to the Internal Medicine Clerkship; however, patient satisfaction was not specifically addressed [2]. 

Social determinants of health (SDH) impact health outcomes in multiple ways, and these disparities disproportionately impact Black Americans [11]. The COVID-19 pandemic threw these disparities into sharp relief, resulting in an executive directive signed by the Governor of the State of Michigan in August of 2020, “Addressing Racism as a Public Health Crisis” [11]. In our practice, approximately 70% of our patients self-identify as Black or African American, and most live in zip codes surrounding the health center, which have historically been made socially vulnerable [12]. Nearly half of our patients are on Medicaid, and just over a quarter are on Medicare. To address social determinants of health, they must first be identified, then the goal of affecting positive change to health outcomes can begin. An integral part of the telemedicine visits performed was the use of an SDH screening tool, developed by a content expert and performed by a medical student at the outset of each visit [2]. This allowed for the identification of a patient's social needs, especially important during the early days of the COVID-19 pandemic when social isolation was becoming a norm. 

With the increased implementation of telemedicine and rapid acceleration of its teaching in medical education due to the COVID-19 pandemic, assessing patient satisfaction with the virtual healthcare they receive is crucial. This study aimed to assess patient satisfaction with the comprehensive care delivered via telemedicine at a university affiliated primary care resident training clinic site in Detroit.

## Materials and methods

Details of the telemedicine visits themselves are reviewed by Abraham et al., including the provision for the delivery of the SDH screening tool at the outset of each visit [2]. The research team process mapped the telemedicine visit and developed questions to assess satisfaction with each component. The survey was reviewed by a content expert with experience in survey methodology. The survey was then piloted for usability internally, and improvements were made, including the plan for survey completion by study personnel over the phone if a patient was unable to complete it online. The final survey consisted of 20 questions, grouped as follows: demographic data (age and gender,) presence of previous experience with telemedicine and/or the technology used (Zoom, San Jose, USA) satisfaction, with components of the virtual visit using a 5-point Likert scale, e.g., 5-Strongly Agree to 1-Strongly Disagree, including assessments of the visit experience, the SDH screen, and the technical aspects of the encounter, and ended with free-text opportunities to describe when telemedicine would or would not be preferred over an in-office visit with a doctor. 

The study was approved by the Wayne State University Institutional Review Board, in tandem with Detroit Medical Center oversight, on June 11^th^, 2020. Data collection began in July 2020. We retrospectively surveyed 167 patients who received telemedicine services between March and July of 2020 at the General Medicine Ambulatory Practice (GMAP) clinic site in Detroit, Michigan, a university-affiliated primary care residency training clinic. Members of the research team contacted patients using the HIPAA-compliant Doximity dialer application and used a script to introduce themselves before conducting the survey. The formal script for the patient outreach can be found in the supplemental materials. Patients were provided the option to have the survey emailed to them for online completion or to complete the survey over the phone with the researcher. All survey responses were recorded in Qualtrics (Utah, USA), and survey data were downloaded directly from the Qualtrics application. With the help of the Wayne State University Research Design and Analysis Unit, results were interpreted through Qualtrics and IBM Corp. Released 2021. IBM SPSS Statistics for Windows, Version 28.0. Armonk, NY: IBM Cor using a linear regression test with p<0.05. 

## Results

Survey response data

Of the 167 patients contacted, 79 completed the “GMAP Telehealth Patient Satisfaction Survey” in its entirety, representing a 47% completion rate. There were no incomplete assessments. Patient demographics revealed that 66% identified as female and 34% as male. Age at the time of the visit in increments of decades was reported, and responses were distributed between two ranges: 62% of participants were aged between 18 and 54 years, and 38% were aged 55 years or more. 

For 81% of respondents, this was their first telemedicine experience, and 19% had previous experience with telemedicine. Prior Zoom (San Jose, USA) application experience was reported by 42% of participants, and 61% of participants found the Zoom platform extremely easy to use. Table 1 depicts the results of the Likert-scaled questions. The ability to hear the physician well during the visit was assessed to be important, and 64% of respondents “strongly” agreed that they could hear their doctor well enough, while 29% “somewhat” agreed. Lastly, 78% of participants reported no technical difficulties, and only 8% of participants reported either “a lot” or “a great deal” of difficulty (Table 1). 

**Table 1:** Survey results according to the Likert scale

Question	Strongly Agree (5)	Somewhat Agree (4)	Neutral (3)	Somewhat Disagree (2)	Strongly Disagree (1)	Mean	
All concerns were addressed	67%	23%	5%	4%	1%		4.51
Online visit met medical care needs	58%	28%	5%	4%	5%		4.30
Able to hear doctor	64%	30%	2%	4%	0%		4.54
	Extremely comfortable (5)	Somewhat comfortable (4)	Neutral (3)	Somewhat Uncomfortable (2)	Extremely Uncomfortable (1)		Mean
Comfort sharing details	72%	20%	4%	4%	0%		4.61
	Definitely Yes (5)	Probably Yes (4)	Maybe (3)	Probably Not (2)	Definitely Not (1)		Mean
Willing to do telemedicine again	51%	30%	8%	10%	1%		4.19
Comfortable using telemedicine	39%	17%	26%	11%	7%		3.67
Comfortable using telemedicine with unfamiliar doctor	21%	37%	15%	22%	5%		3.46
	Extremely Easy (5)	Somewhat Easy (4)	Neutral (3)	Somewhat Difficult (2)	Extremely Difficult (1)		Mean
ZOOM was easy to use	61%	22%	10%	6%	1%		4.33
	None at all (5)	A little (4)	Moderate amount (3)	A lot (2)	A great deal (1)		Mean
Technical difficulties with visit	78%	13%	1%	5%	3%		4.59
	Excellent (5)	Good (4)	Neutral (3)	Fair (2)	Poor (1)		Mean
Overall quality of care	53%	31%	8%	8%	0%		4.30

Regarding comfort in sharing details about patient concerns, 72% of participants were “extremely comfortable,” and approximately 4% were “somewhat uncomfortable.” Of note, all the patients who reported discomfort sharing details over telehealth (n=3) were over the age of 85 years. With respect to addressing patient concerns in a virtual format, 67% of survey participants stated that they “strongly agreed” with the statement “I feel all of my concerns were addressed,” while only 5% “somewhat” or “strongly disagreed.” Furthermore, 58% of participants “strongly agreed” that the visit met their medical care needs (Table 1).

Survey participants were assessed on comfort seeing a physician via telemedicine they had not previously met, self-perceived quality of care delivered, and willingness to do telemedicine again. About 21% of respondents “definitely” felt comfortable doing a telemedicine visit with a doctor they had never met, with only 5% “definitely not” comfortable. Regarding the overall quality of care the patient received through telemedicine, 85% reported they received “good” or “excellent” care. Over 80% of participants surveyed stated they would “definitely” or “probably” be willing to do another telemedicine visit, with only 11% stating they would “probably” or “definitely” not (Table 1). 

All survey participants were asked if their doctor addressed their social concerns during the visit, and over half (54%) of respondents either “somewhat” or “strongly” agreed. Resources to assist with social concerns were provided in 35% of encounters, and 93% of those patients agreed “somewhat” or “strongly” that the resources provided were helpful (Table 2).

**Table 2:** Social concerns survey results

Question	Strongly Agree (5)	Somewhat Agree (4)	Neutral (3)	Somewhat Disagree (2)	Strongly Disagree (1)	Mean
Doctor addressed social concerns	63%	27%	8%	2%	0%	4.51
Resources for social concerns provided were helpful	59%	18%	23%	0%	0%	4.36
	Yes (2)	No (1)	Does not apply (0)			Mean
My doctor provided me resources for social concerns	35%	5%	60%			0.76

The final two questions left space for free text responses covering reasons for visits for which telemedicine may not be or would never be preferred. Many respondents cited the need for labs and/or imaging that cannot be obtained via telemedicine. Some mentioned a simple desire for in-person interaction. Others affirmed their desire to at least visit in-person with their physician periodically. 

Regression/Association analysis

Patient satisfaction was based on patients’ responses to survey questions “overall quality of care received” and “willingness to do telemedicine again.” There was a statistically significant correlation between the overall perceived quality of care received and all concerns addressed (p < 0.001), as well as the visit meeting their medical needs (p < 0.001). There was also a statistically significant correlation between willingness to do telemedicine again and all concerns addressed (p < 0.001), as well as the visit meeting their medical needs (p < 0.001). Perceived quality of care was compared against male and female gender (p = 0.495) and age groups over 55 and under 54 (p = 0.702) using a t-test with a p-value <0.05. There was no statistically significant difference in willingness to do telemedicine again, and both gender (p < 0.478) and age groups (p < 0.221). There was a statistically significant correlation between willingness to do telemedicine again and patient comfort in sharing medical concerns (p = 0.005). Patients were asked if the Zoom platform was easy to use; however, the reported ease of use was not significantly correlated with an improvement in overall quality of care and willingness to do telemedicine again (p = 0.252). 

## Discussion

This study evaluates patient satisfaction with the care delivered by their physician via telemedicine and whether the patient felt their medical and social needs were adequately met virtually. We found that most respondents were satisfied with the overall care delivered during the telemedicine visit. Although most patients had no previous experience with telemedicine, we found that the majority were satisfied and comfortable with the virtual visit format and would be willing to engage in future telemedicine visits. The high patient satisfaction rates with telemedicine we observed are consistent with previously reported rates in other studies [13]. 

Unlike similar studies evaluating patient satisfaction with telemedicine, we assessed the success of addressing not only the medical but also the social needs of patients via the virtual format. This study took place at an urban clinical center were addressing the social needs of patients continues to be vitally important in the overall improvement of health outcomes and patient well-being. Previous studies have found that challenges with telemedicine services are more profound in patients who are socioeconomically disadvantaged or who have learning or physical disabilities [14]. Our practice serves many of these socially vulnerable patients. Most of our patients reported having their medical needs met. Though the specific social needs of these survey respondents were not collected, the prompt during their telemedicine visit included possible difficulty with paying bills, access to food, clothing, housing, obtaining needed medication, mental health concerns, transportation, childcare, or other such concerns [2]. Those who self-reported such concerns found that their needs were adequately addressed during the telemedicine visit, and if resources were provided, they were almost always reported to be helpful. While addressing social needs virtually is more nuanced, the addition of this component of medical care to the telemedicine visit allows for a thorough patient evaluation. 

The use of telemedicine and virtual healthcare visits became integral during the early days of the COVID-19 pandemic, as patients needed care but were either unable to be seen face-to-face or limited by the concern for virus exposure and transmission [15]. This is still relevant today, and studies continue to emerge describing how the expanded use of telemedicine provides an avenue for engaging vulnerable patients previously excluded from care [16]. While the integration of telemedicine allows for convenient access to medical care, assuring quality care and patient satisfaction is important. As technological advances in virtual physical exam tools, video visits, and internet connectivity continue to improve and expand, so will the access to medical care via virtual format. The limitations of telemedicine, including the concern for data privacy and security, are currently being processed and addressed [14]. Importantly, the development of a robust strategy to address social concerns via telemedicine must continue to provide satisfactory and thorough patient care and achieve health equity. 

Limitations to the generalizability of our study include that it involved a single site. Though self-identified race/ethnicity was not collected as part of the demographic data for this survey, this clinic site serves primarily patients who self-identify as Black or African American, and the results may not apply to other patient populations. A response rate of over 50% is reasonable for survey studies [17], and ours fell just short of this. Another limitation is our sample size. Patients were contacted via phone or email several weeks after the telemedicine visit, which creates the potential for recall bias within the results. Follow-up studies are indicated to assess whether this intervention improved patient care outcomes.

## Conclusions

This study evaluated multiple dimensions of patient satisfaction with a telemedicine visit instead of an in-office visit during the early days of the COVID-19 pandemic. Telemedicine was well received, with high patient satisfaction for addressing medical and social concerns. The results of this study support the use of telemedicine to assess social determinants of health in an underserved minoritized patient population. This clinical site has integrated telemedicine into its long-term daily operations since the inception of this survey. Future studies are planned to assess whether patient satisfaction with telemedicine impacts health outcomes. 

## Appendices

Script for calls 

Researcher: “Hello, I am a medical student calling from Dr. (Their PCP)’s office looking to speak with (Patient name).” 

Patient Response: “This is he/she speaking” 

Researcher: I just wanted to touchbase with you and ask a couple of questions regarding your recent telehealth meeting with Dr. (Their PCP) on the (Date of meeting). Do you have a few moments?” 

Patient Response: “Yes, go ahead” 

Researcher: “First, I wanted to check in with you. Since then, how have you been doing?” 

Patient Response: If posing a concern redirect them to contact their PCP about the issue; if they are stating they are doing well, reinforce and move forward with script. 

Researcher: “Since you are part of the first group of individuals that participated in a telehealth office visit in which we discussed things through video chat or over the phone, I was hoping to get some feedback from you. Would you be willing to answer a short 5-minute survey? I can send it to you through email or even ask you the questions over the phone right now, if you have the time.” 
